# Post-mortem analyses of PiB and flutemetamol in diffuse and cored amyloid-β plaques in Alzheimer’s disease

**DOI:** 10.1007/s00401-020-02175-1

**Published:** 2020-08-09

**Authors:** Milos D. Ikonomovic, Christopher J. Buckley, Eric E. Abrahamson, Julia K. Kofler, Chester A. Mathis, William E. Klunk, Gill Farrar

**Affiliations:** 1 Geriatric Research Education and Clinical Center, VA Pittsburgh Healthcare System, Pittsburgh, PA USA; 2 Department of Neurology, University of Pittsburgh, Pittsburgh, PA USA; 3 Department of Psychiatry, University of Pittsburgh, Pittsburgh, PA USA; 4 Department of Pathology, University of Pittsburgh, Pittsburgh, PA USA; 5 Department of Radiology, University of Pittsburgh, Pittsburgh, PA USA; 6 GE Healthcare, Amersham, UK; 7 University of Pittsburgh School of Medicine, Thomas Detre Hall of the WPIC, Room 1421, 3811 O’Hara Street, Pittsburgh, 15213-2593 PA USA

**Keywords:** Amyloid, Flutemetamol, Fluorescence microscopy, Pittsburgh compound B, Positron emission tomography, Striatum

## Abstract

Specificity and sensitivity of positron emission tomography (PET) radiopharmaceuticals targeting fibrillar amyloid-β (Aβ) deposits is high for detection of neuritic Aβ plaques, a mature form of Aβ deposits which often have dense Aβ core (i.e., cored plaques). However, imaging-to-autopsy validation studies of amyloid PET radioligands have identified several false positive cases all of which had mainly diffuse Aβ plaques (i.e., plaques without neuritic pathology or dense amyloid core), and high amyloid PET signal was reported in the striatum where diffuse plaques predominate in Alzheimer’s disease (AD). Relative contributions of different plaque types to amyloid PET signal is unclear, particularly in neocortical areas where they are intermixed in AD. In vitro binding assay and autoradiography were performed using [^3^H]flutemetamol and [^3^H]Pittsburgh Compound-B (PiB) in frozen brain homogenates from 30 autopsy cases including sporadic AD and non-AD controls with a range of brain Aβ burden and plaque density. Fixed tissue sections of frontal cortex and caudate from 10 of the AD cases were processed for microscopy using fluorescent derivatives of flutemetamol (cyano-flutemetamol) and PiB (cyano-PiB) and compared to Aβ immunohistochemistry and pan-amyloid (X-34) histology. Using epifluorescence microscopy, percent area coverage and fluorescence output values of cyano-PiB- and cyano-flutemetamol-labeled plaques in two-dimensional microscopic fields were then calculated and combined to obtain integrated density measurements. Using confocal microscopy, we analysed total fluorescence output of the entire three-dimensional volume of individual cored plaques and diffuse plaques labeled with cyano-flutemetamol or cyano-PiB. [^3^H]Flutemetamol and [^3^H]PiB binding values in tissue homogenates correlated strongly and their binding pattern in tissue sections, as seen on autoradiograms, overlapped the pattern of Aβ-immunoreactive plaques on directly adjacent sections. Cyano-flutemetamol and cyano-PiB fluorescence was prominent in cored plaques and less so in diffuse plaques. Across brain regions and cases, percent area coverage of cyano-flutemetamol-labeled plaques correlated strongly with cyano-PiB-labeled and Aβ-immunoreactive plaques. For both ligands, plaque burden, calculated as percent area coverage of all Aβ plaque types, was similar in frontal cortex and caudate regions, while integrated density values were significantly greater in frontal cortex, which contained both cored plaques and diffuse plaques, compared to the caudate, which contained only diffuse plaques. Three-dimensional analysis of individual plaques labeled with either ligand showed that total fluorescence output of a single cored plaque was equivalent to total fluorescence output of approximately three diffuse plaques of similar volume. Our results indicate that [^18^F]flutemetamol and [^11^C]PiB PET signal is influenced by both diffuse plaques and cored plaques, and therefore is likely a function of plaque size and density of Aβ fibrils in plaques. Brain areas with large volumes/frequencies of diffuse plaques could yield [^18^F]flutemetamol and [^11^C]PiB PET retention levels comparable to brain regions with a lower volume/frequency of cored plaques.

**Electronic supplementary material:**

The online version of this article (10.1007/s00401-020-02175-1) contains supplementary material, which is available to authorized users.

## Introduction

Development of positron emission tomography (PET) radiopharmaceuticals targeting fibrillar amyloid-β (Aβ) has enabled imaging of Aβ deposits (plaques) in brains of living people clinically suspected of having Alzheimer’s disease (AD). The National Institute of Neurological and Communicative Disorders and Stroke and the Alzheimer's Disease and Related Disorders Association criteria [29] from over three decades ago permitted a clinical diagnosis confirmed only at post-mortem and has evolved into a sophisticated construct of defining AD as a pathological continuum from the preclinical state via a prodromal AD intermediate to dementia due to AD [10]. Regarding imaging biomarkers, the latest concept is a “research framework” which combines Aβ and tau measures with measures of neurodegeneration [20] to assign subjects and patients into groups by biomarker status. However, relevant thresholds of amyloid PET signal for normality and abnormality remain to be determined [19, 39] and require further research of amyloid PET imaging and other biomarkers such as cerebral spinal fluid (CSF) measures in relation to underlying pathology.

[^11^C]PiB PET has been used widely to image Aβ deposition and progression in research settings since 2003 [22, 26] including studies of large cohorts such as Wisconsin Registry for Alzheimer’s Prevention (WRAP) [37], Harvard Aging Brain Study (HABS) [9], Mayo Clinic Study of Aging (MCSA) [35], Baltimore Longitudinal Study of Aging (BLSA) [34], Australian Imaging Biomarkers and Lifestyle study (AIBL) [11], Dominantly Inherited Alzheimer’s Network (DIAN) [2], and Neurodegeneration in Aging Down syndrome (NiAD) [42] among others. Although [^11^C]PiB PET is somewhat more superior to other recently-developed PET ligands for Aβ plaques in its sensitivity to detect most, if not all Aβ plaque forms [17], it is limited by the need for an on-site cyclotron for ^11^C-labeling. The development of Aβ binding agents labeled with longer living fluorine-18, including [^18^F]PiB (flutemetamol/Vizamyl™ [8, 43]), florbetapir (Amyvid™ [7, 47]), and florbetaben (Neuraceq™ [1, 36]) offers an opportunity for assessment of Aβ plaques in a clinical setting. These three Food and Drug Administration (FDA)-approved PET radiopharmaceuticals for imaging of Aβ plaques were evaluated for sensitivity and specificity of visual inspection of retention maps against the presence of neuritic Aβ plaques due to the central role of these lesions in neuropathology criteria for AD defined by the Consortium to Establish a Registry for Alzheimer’s Disease (CERAD) [30] and National Institute on Aging-Reagan Institute [41]. These performance measures of PET amyloid radiopharmaceuticals showed a high accuracy [6, 8, 13, 16, 36, 38], supporting the assumption that neuritic Aβ plaques, which often have cores of densely-packed Aβ fibrils, strongly influence amyloid PET signal. Near the time the FDA-approved ^18^F-labeled Aβ ligands, neuropathological criteria for AD were updated by the National Institute on Aging-Alzheimer's Association (NIA-AA) [14, 31] to include both CERAD criteria for scoring of neuritic Aβ plaques [30] and Thal phases which rely on immunohistochemical detection of all types of Aβ plaques [40]. Using the 2012 NIA-AA criteria as the standard of truth resolved false positive read cases for the PET tracer [^18^F]flutemetamol that were encountered using CERAD scoring alone [16], confirming the need for inclusion of all types of Aβ plaques in pathological assessment of amyloid PET tracers. A better understanding of the contribution of different Aβ plaque types to amyloid PET ligand retention is now of upmost importance as the field progresses to using regional uptake measures [12, 28] to characterize the initial and later phases of Aβ deposition as well as exploring the possibilities that regional measures can be used to predict possible changes in clinical state [32, 40].

In AD brain, neuritic Aβ plaques are abundant in neocortex and often contain cores of densely-packed Aβ fibrils [14, 31]. Some brain regions which lack significant amounts of neuritic and cored Aβ plaques, such as the striatum [5], also show strong amyloid PET signal in sporadic and familial AD [4, 17, 18, 23, 25, 44], likely reflecting the high density of diffuse Aβ plaques in this area. Recently, it was reported that [^18^F]flutemetamol PET detects moderate-to-frequent diffuse Aβ plaques in the striatum and this could be used to improve the accuracy of clinicopathological diagnosis of AD [4]. These studies underscore the need for elucidating the contribution of different Aβ plaque types to retention of amyloid PET radiopharmaceuticals. To this end, the current study used [^3^H]flutemetamol and [^3^H]PiB for tissue binding studies, and cyano-labeled flutemetamol (cyano-flutemetamol) and PiB (cyano-PiB) for histofluorescence studies to explore the degree to which diffuse and cored types of Aβ plaques contribute to the total binding of flutemetamol and PiB in autopsy brain tissues.

## Methods

Studies were approved by the University of Pittsburgh Committee for Oversight of Research and Clinical Training Involving Decedents. Brain tissue samples were obtained from 30 autopsy cases in the University of Pittsburgh Alzheimer’s Disease Research Center’s brain bank. Cases identified neuropathologically as AD as well as non-AD controls with none or sparse amyloid pathology were selected from this series based on availability of tissue for studying. Case demographics and neuropathological characteristics are listed in Table 1. Cases had no known presenilin 1 mutations or cotton wool plaques and were therefore considered as sporadic AD.

**Table 1:** Case demographic and neuropathological characteristics

Case #	PMI (h)	Age (y)	Brain weight (g)	Sex	APOE	Braak NFT stage	CERAD criteria diagnosis	NIA-Reagan criteria diagnosis (likelihood of AD)	CAA	Primary neuropathology diagnosis	History of dementia
1	5	53	1145	M	n.d	0	Normal	N/A	None	Normal	No
2	4	82	1060	F	n.d	I	Normal	N/A	None	PART, possible (sparse diffuse plaques)	No
3	10	75	1150	M	n.d	I	Normal	N/A	None	PART, possible (sparse diffuse plaques)	No
4	4.5	81	1030	F	n.d	II	Possible AD	Low	Mild	Multi-infarct dementia with sparse neuritic plaques	Yes
5	9	72	1120	F	34	II	Possible AD	N/A	None	AD pathology (sparse neuritic plaques)	No
6	3	79	1370	M	34	II	Definite AD	Low	Moderate	DLB, neocortical	Yes
7	3	89	1090	F	33	III	Definite AD	Intermediate	Moderate	AD	Yes
8	5	83	1170	F	33	III	Definite AD	Intermediate	Mild	AD	Yes
9	5	87	1250	M	33	IV	Definite AD	Intermediate	Mild	AD	Yes
10	8	75	1260	M	33	IV	Definite AD	Intermediate	Severe	DLB, neocortical	Yes
11	7	86	1170	M	33	V	Definite AD	High	Mild	AD	Yes
12	9	57	1250	M	34	V	Definite AD	High	Mild	AD	Yes
13	4.5	87	1080	F	34	VI	Definite AD	High	Severe	AD	Yes
14	7	57	1250	M	44	VI	Definite AD	High	Severe	AD	Yes
15	4	78	1250	M	44	VI	Definite AD	High	Mild	AD	Yes
16	4.5	80	1110	F	33	VI	Definite AD	High	Mild	AD	Yes
17	4	79	910	F	34	VI	Definite AD	High	Severe	AD	Yes
18	8	89	1080	F	33	VI	Definite AD	High	None	AD	Yes
19	10	82	1225	F	33	VI	Definite AD	High	Mild	AD	Yes
20	3	77	1180	F	23	VI	Definite AD	High	Severe	AD	Yes
21	3	75	1080	F	33	VI	Definite AD	High	Severe	AD	Yes
22	9	85	950	F	33	VI	Definite AD	High	Mild	AD	Yes
23	11	64	1070	F	34	VI	Definite AD	High	Mild	AD	Yes
24	10	61	1160	M	44	VI	Definite AD	High	Severe	AD	Yes
25	14	81	1230	M	33	VI	Definite AD	High	None	AD	Yes
26	4	69	1140	F	34	VI	Definite AD	High	Mild	AD	Yes
27	10	83	1300	M	33	VI	Definite AD	High	Severe	AD	Yes
28	8	83	1120	F	34	VI	Definite AD	High	Severe	AD	Yes
29	10	85	1250	M	44	VI	Definite AD	High	Severe	AD	Yes
30	4	77	1200	M	33	VI	Definite AD	High	Mild	AD	Yes

*APOE* apolipoprotein E genotype, *AD* Alzheimer’s disease, *CAA* cerebral amyloid angiopathy, *CERAD* Consortium to establish a registry for Alzheimer’s disease, *DLB* Dementia with Lewy bodies, *F* female, *g* grams, *h* hours, *M* male, *NFT* neurofibrillary tangle, *PART* primary age-related tauopathy, *PMI* post-mortem interval, *y* years

### Brain tissue processing

At autopsy, the left cerebral hemisphere was placed in ice-cold 4% paraformaldehyde (made in 0.01 M sodium phosphate buffer, pH 7.4) for 21 days. CERAD-designated brain regions were sampled for diagnostic purposes [30]. Fixed tissue blocks containing cortical and subcortical areas (including striatum) were immersed sequentially in 15% and 30% sucrose made in sodium phosphate buffer and then sliced on a freezing, sliding microtome (model 860, American Optical Corporation, Buffalo, NY) into 40 µm thick sections for epifluorescence studies of all regions and into 90–160 µm thick sections for confocal microscopy studies of striatum and frontal cortex. Tissue sections were collected serially in cryoprotectant solution [45] and stored at − 20 °C. Prior to histological processing, tissue sections were rinsed in 0.01 potassium phosphate buffer (pH 7.4), mounted onto charged slides, air-dried for 15 min, and kept at room temperature until used. One portion of the tissue block was embedded in paraffin and sliced serially into 8 µm thick sections. Prior to processing, paraffin was removed by sequential immersion of sections in xylenes and ethanol. Sections were then rehydrated, washed in potassium phosphate buffer, air-dried for 15 min, and kept at room temperature until used. Frozen tissue was obtained post-mortem from the right hemisphere and stored at − 80 °C until assayed.

### Cyano-PiB and cyano-flutemetamol histofluorescence

Cyano-PiB (2-(4′-methylaminophenyl)-6-cyanobenzothiazole) is a highly fluorescent derivative of PiB (2-(4′-methylaminophenyl)-6-hydroxybenzothiazole) created by replacing the 6-hydroxy group of PiB with a 6-cyano group (provided by W.E.K. and C.A.M.) [27]. When applied to fixed tissue sections, cyano-PiB labels Aβ deposits in neuropil plaques and in the cerebrovasculature [17]. Cyano-flutemetamol (2-(3′-fluoro-4′-methylaminophenyl)-6-cyanobenzothiazole) is a highly fluorescent derivative of flutemetamol (2-(3′-fluoro-4′-methylaminophenyl)-6-hydroxybenzothiazole) which itself is a structural analog of PiB (provided by W.E.K. and C.A.M.) [33]. Histofluorescence procedures were performed identically on both 4% paraformaldehyde-fixed sections and formalin-fixed, paraffin-embedded tissue sections. After rinsing in potassium phosphate buffer, sections were incubated in 10 µM cyano-PiB or 10 µM cyano-flutemetamol for 45 min in dark conditions at room temperature. Sections were then dipped three times in potassium phosphate buffer, incubated for 1 min in fresh potassium phosphate buffer, and coverslipped with Fluoromount (SouthernBiotech, Birmingham, AL).

### Epifluorescence studies

Cyano-labeled compounds were viewed using an Olympus BX53 microscope with fluorescent attachment (X-cite 120Q) and equipped with an Olympus DP72 digital camera connected to a Dell Precision T5500 computer running Olympus cell Sens Standard 1.6 imaging software. Cyano fluorescence was visualized using a hydroxycoumarin filter (excitation peak: 405 nm, beam splitter: 425 nm, emission peak: 460 nm; #31016, Chroma, Bellows Falls, VT) and images were obtained using a 10 × microscope objective (infinity-corrected, numerical aperture = 0.4; Olympus). Fluorescence imaging and image acquisition parameters were held constant throughout the experiment. Consistency in these parameters across imaging sessions was confirmed by imaging a slide containing fluorescent microspheres (SPHERO Rainbow, Spherotech, Inc, Lake Forest, IL). A total of nine images were obtained from three sections (three images per section) for each case/region, focusing on the gray matter. Each image was saved as a tagged image format file and imported into ImageJ (Rasband WS, ImageJ, US National Institutes of Health, Bethesda, Maryland) for analysis. For each microscopic field, plaque load was calculated by measuring the percent area occupied by plaques relative to total area measured to give a value of percent area coverage. Cerebral vascular Aβ deposits were only rarely encountered and were excluded from the analysis. In the caudate and frontal cortex from 10 AD cases, integrated fluorescence intensity values were obtained using the Integrated Density option from the Analyze menu of ImageJ.

After epifluorescence microscopic imaging, subsets of cyano-flutemetamol-processed 4% PFA-fixed, 40 µm thick sections were de-coverslipped in tap water, rinsed in potassium phosphate buffer, and photobleached prior to overstaining. Sections were then overstained using cyano-PiB as described above, images were acquired from the same microscopic fields from which cyano-flutemetamol images were obtained, and sections were de-coverslipped and photobleached a second time. Photobleached sections were then overstained a second time using X-34, a highly fluorescent derivative of Congo red and pan-amyloid marker [15] to detect Aβ in parenchymal and vascular deposits as well as tau accumulates in neuritic pathology. X-34 has an absorption peak at 367 nm and emission peak at 497 nm [15] and was visualized using a violet filter (excitation peak: 405 nm, beam splitter: 440 nm, emission peak: 455 nm; #11005, Chroma) Sets of four serial paraffin-embedded sections (thickness: 8 µm) were processed for (1) cyano-flutemetamol, (2) Aβ immunohistochemistry, (3) cyano-PiB, and (4) Bielschowsky silver stain histology [46]. Sections processed for Aβ immunohistochemistry were pretreated with 90% formic acid for 2 min, incubated in antibody clone 4G8 (Covance #SIG39220) at 1:500 dilution overnight at 4 °C, followed by a 2-h incubation in rabbit anti-mouse biotinylated secondary antibody (Jackson ImmunoResearch, West Grove, PA) and avidin–biotin chemistry using Nova Red (Vector) with hematoxylin counterstain.

### Confocal microscopy studies

Slide-mounted tissue sections (thickness 90–160 µm) from frontal cortex and caudate were rinsed in potassium phosphate buffer and processed using cyano-PiB and cyano-flutemetamol as described above. Image acquisition was performed using an Olympus BX-51WI upright microscope equipped with an Olympus DSU spinning disk confocal, a super-corrected 60 × Olympus Plan Apo N 1.42 numerical aperture oil immersion objective, MBF CX9000 front-mounted digital camera (MBF Bioscience, Williston, VT), a BioPrecision2 XYZ motorized stage with linear XYZ encoders (Ludl, Hawthorne, NY), a Sedat Quad 89,000 filter set (Chroma, excitation peak: 402 nm, beam splitter 425 nm, emission peak: 455 nm), and a Lumen 220 metal halide lamp (Prior Scientific, Rockland, MA). The microscope was controlled using Stereo Investigator 11 (MBF Bioscience) and SlideBook 6 (Intelligent Imaging Innovations, Denver, CO) software. All images were obtained using the acquisition parameters: 1024 × 1024 frame size, near isotropic pixels (*z* axis: 0.11 µm; *x* and *y* axis: 0.108 µm), 0.11 µm *z* axis step size, and 100 ms exposure time. The objective of this experiment was to obtain confocal stacks through the complete volume of cored plaques (including both the core and the corona) and diffuse plaques, thus plaques were sampled from the inner third of the tissue section *z* axis to avoid clipping of plaques at the tissue surfaces. Obtaining the full volume of plaques was achieved most readily using 160 microns-thick tissue sections thus tissue sections of this thickness were used for the quantitative analysis. Tissue shrinkage during processing was minimal and did not appear to affect the morphology of plaques—for example, the *x*–*y* diameter of a cored plaque circular in the *x*–*y* plane equaled its *z* axis diameter. Plaques within confocal stacks were analysed as three-dimensional volumes. Once a three-dimensional stack was created, the analysis methodology consisted of selecting a region outside the plaque diameter (background) in the mid-plane and subtracting this from all voxel values within the plaque. A voxel in the *x*, *y*, and *z* dimensions was positioned at the centre of the plaque and a spherical volume of interest was then grown radially outward to the periphery of each plaque and the net integrated intensity and volume were measured such that the total fluorescent output could be obtained together with the fluorescence intensity per unit volume.

### [^3^H]Flutemetamol and [^3^H]PiB autoradiography on tissue sections

A fresh (unfixed) block of tissue containing the hippocampus was dissected at autopsy and immediately frozen on dry ice and stored at − 80 °C until processing. The block was then cut using a cryostat (Reichert Jung Frigocut 2800 E, Mannheim, Germany) at − 20 °C into sequential, 10 µm thick sections. Sections were immediately thaw-mounted onto Histobond + slides (Marienfeld-Superior, Lauda-Königshofen, Germany) and stored at − 80 °C. Slide-mounted tissue sections were immersed in sodium phosphate buffer (pH 7.4) for 30 min, followed by incubation for 1 h at room temperature in ^3^H-labeled compound (3 nM), rinsed in sodium phosphate buffer at 4 °C, air dried, and placed in an exposure cassette (#29-1755-23, GE Healthcare Bio-Sciences AB, Uppsala, Sweden) in close apposition to a storage phosphor screen (BAS-IP TR 2025 tritium screen, Fujifilm Corp, Tokyo, Japan) for six days. The phosphor screen was scanned using a Typhoon FLA7000 imager (GE Healthcare) equipped with a 390 nm band-pass imaging plate filter and images were converted to tagged image format files for analysis. Sections immediately adjacent to those used for autoradiography were processed for immunofluorescence using anti-Aβ antibody clone 6E10 (sections incubated in ice-cold 4% paraformaldehyde for 15 min, pretreated with 90% formic acid pretreatment for 2 min and incubated in 6E10 antibody at 1:250 dilution overnight at 4ºC; BioLegend #803002, lot #B1198896; [21]) and an affinity purified anti-mouse secondary antibody conjugated to the Alexa594 fluorophore (dilution = 1:250 from 1.5 mg/ml stock; Jackson ImmunoResearch #115–585-146, lot #122683). The Alexa594 fluorophore has a peak absorption band at 591 nm and peak emission band at 614 nm and was detected using rhodamine filter (excitation peak: 545 nm, beam splitter: 565 nm, emission peak: 605 nm; #49004, Chroma).

### Competition binding assays

To determine the Ki (inhibition binding constant) for PiB, flutemetamol and their cyano-labeled derivatives, binding studies were performed with slight modifications of a procedure previously described in detail [24]. Approximately 1 nM [^3^H]PiB or [^3^H]flutemetamol and the unlabeled compound (over a concentration range of 0.2–1000 nM) were prepared in 900 µl sodium phosphate-buffered saline. Binding was initiated by addition of 100 µl of a 1 mg/ml homogenate of frontal cortex tissue from an AD case in sodium phosphate-buffered saline (in triplicate) and the samples were incubated at 22 °C for 60 min. Filtration was performed as previously described except that the filters were washed three times [24].

### Statistical analyses

Integrated density and plaque load measures in caudate and frontal cortex were compared using Wilcoxon rank-sum test. Correlations were assessed using Spearman rank-order test. These non-parametric statistical tests were used since the data were not normally distributed as determined by the Kolmogorov-Smirnoff test. All statistical tests were performed using GraphPad Prism 7.0 (GraphPad Software, Inc., San Diego, CA).

## Results

### Biochemical validation of [^3^H]flutemetamol and [^3^H]PiB binding to Aβ plaques

**Fig. 1:**
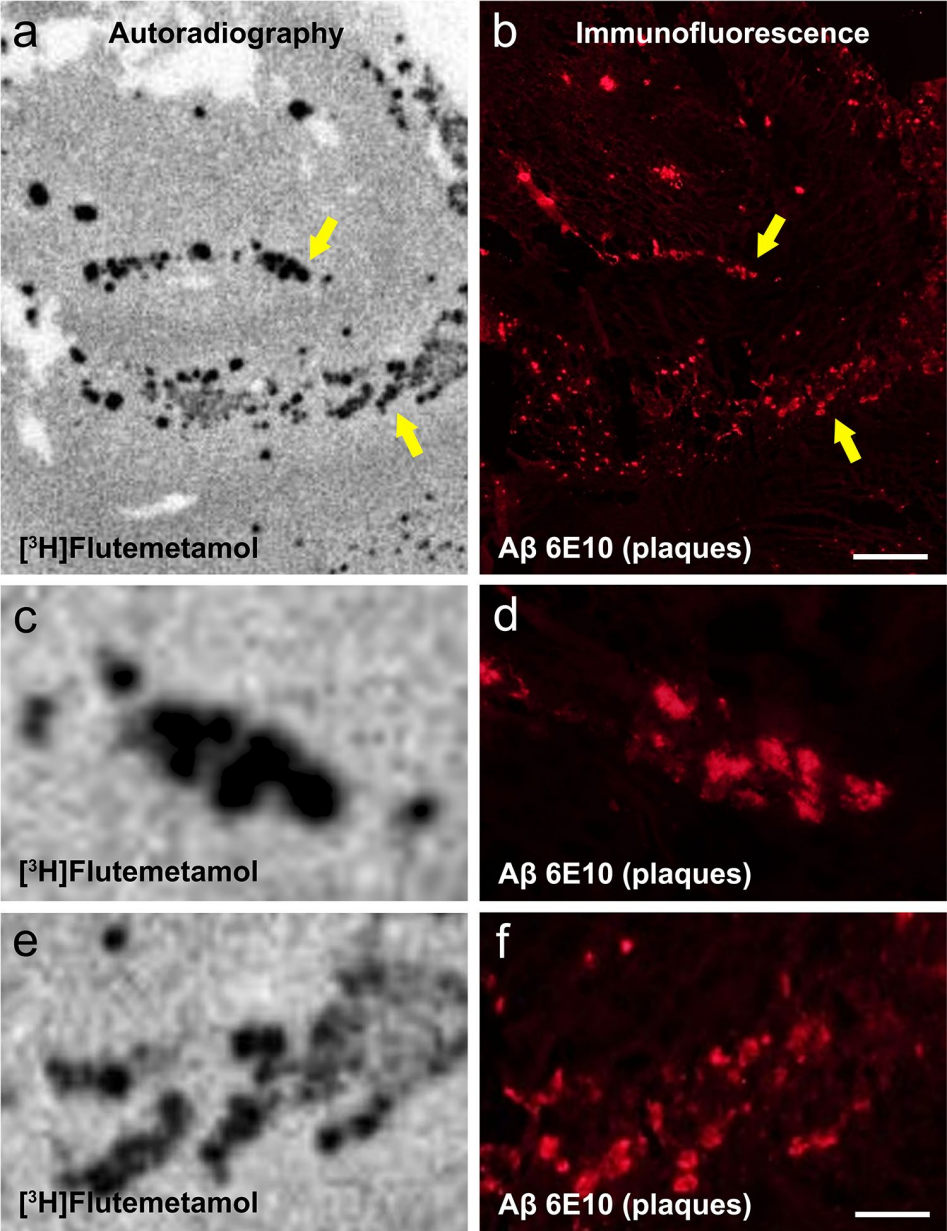
[^3^H]Flutemetamol autoradiography on a frozen, 10 µm section of hippocampus (**a**, **c**, **e**) from case #21 (AD; Table 1) compared to immunofluorescence with anti-Aβ antibody clone 6E10 in an immediately adjacent section (**b**, **d**, **f**) from the same AD case. Arrows in **a** and **b** point at two areas shown at high magnification in paired images **c**, **d** and **e**, **f**. There is high correspondence between [^3^H]flutemetamol autoradiography signal and Aβ-immunofluorescent plaques. Scale bar = 500 µm (**a**, **b**), 50 µm (**c**–**f**)

**Fig. 2:**
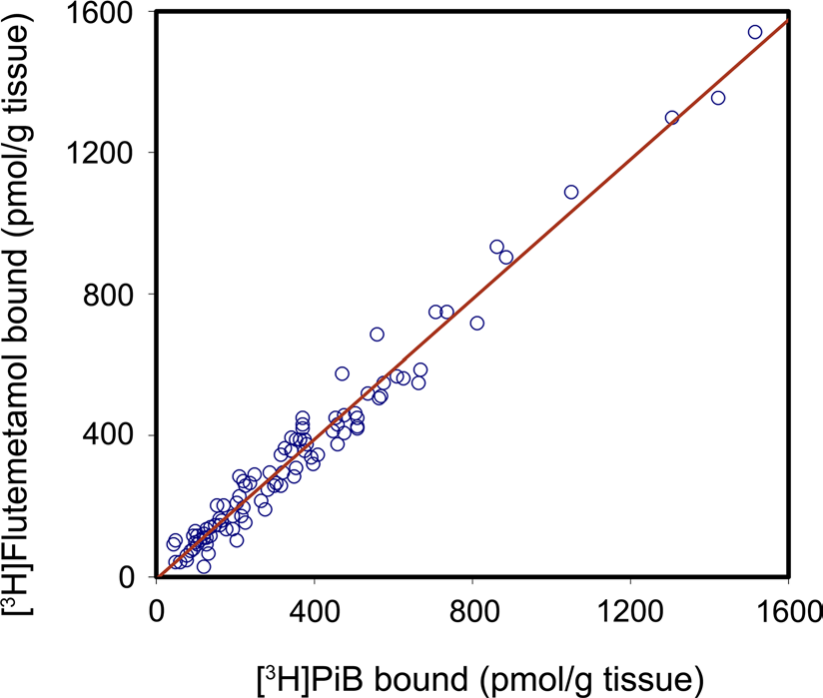
Correlation analysis of [^3^H]flutemetamol and [^3^H]PiB in vitro binding using frozen homogenates of frontal, temporal, and occipital cortex from the 30 autopsy cases (Table 1). There is a strong direct correlation between binding values of the two ligands (*R* = 0.98)

[^3^H]Flutemetamol autoradiography signal matched the distribution of Aβ-immunofluorescent plaques in adjacent sections of AD hippocampus (Fig. 1). Similar results were obtained using [^3^H]PiB (not shown). In frozen AD brain tissue homogenates, there was a strong correlation between [^3^H]flutemetamol and [^3^H]PiB binding using an in vitro binding assay (Fig. 2).

### Histopathology characterization of cyano-flutemetamol and cyano-PiB

**Fig. 3:**
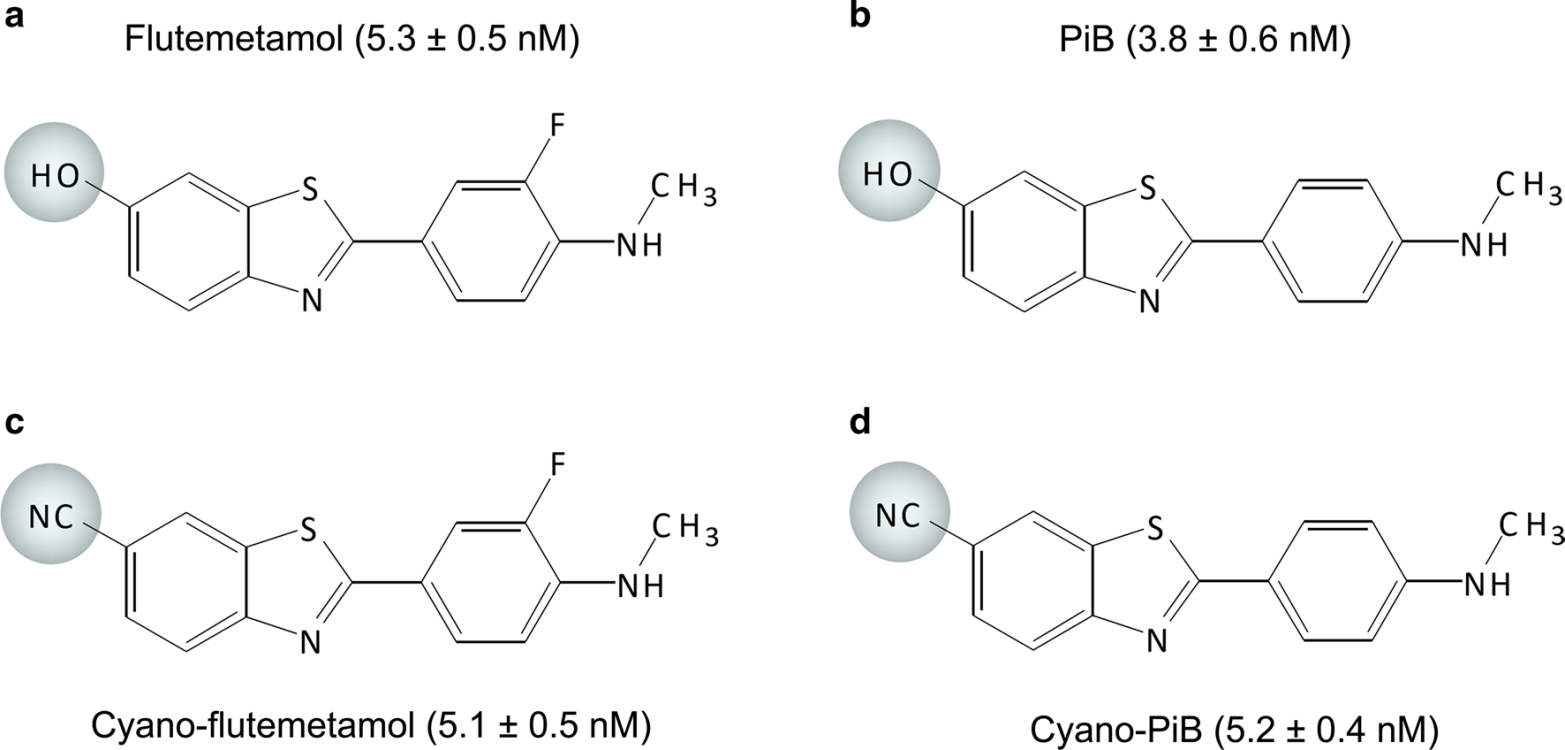
Chemical structure depictions of 2-(3′-fluoro-4′-methylaminophenyl)-6-hydroxybenzothiazole (**a**, flutemetamol), 2-(4′-methylaminophenyl)-6-hydroxybenzothiazole (**b**, PiB), 2-(3′-fluoro-4′-methylaminophenyl)-6-cyanobenzothiazole (**c**, cyano-flutemetamol), and 2-(4′-methylaminophenyl)-6-cyanobenzothiazole (**d**, cyano-PiB). Each compound’s inhibition binding constant determined by competition assay using cortical tissue homogenate from an AD case is provided in parentheses

**Fig. 4:**
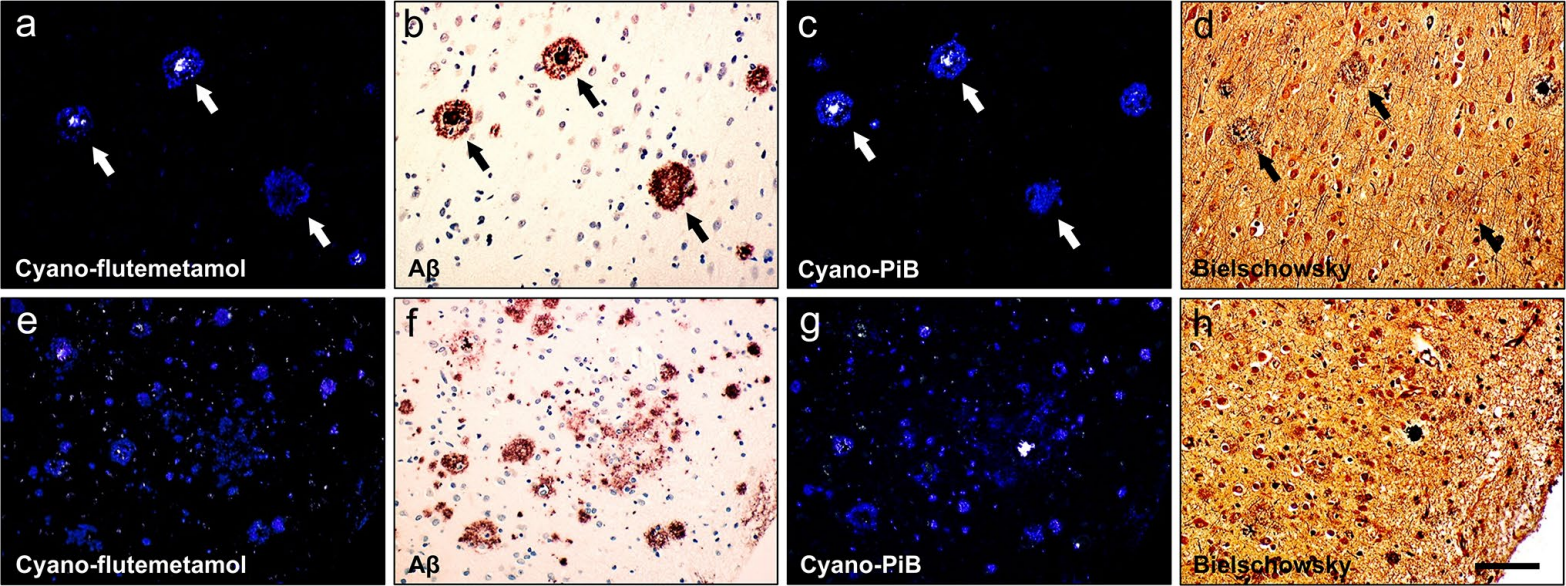
Two sets of sequential paraffin sections (thickness = 8 µm; **a**–**d** and **e**–**h**) of frontal cortex from case #27 (AD; Table 1) processed using cyano-flutemetamol histofluorescence (**a**, **e**), amyloid-β immunohistochemistry (**b**, **f**; antibody clone 4G8), cyano-PiB histofluorescence (**c**, **g**), and Bielschowsky silver stain (**d**, **h**). Arrows in **a**–**d** point to the same plaques labeled with each of the four markers. Scale bar = 100 µm

**Fig. 5:**
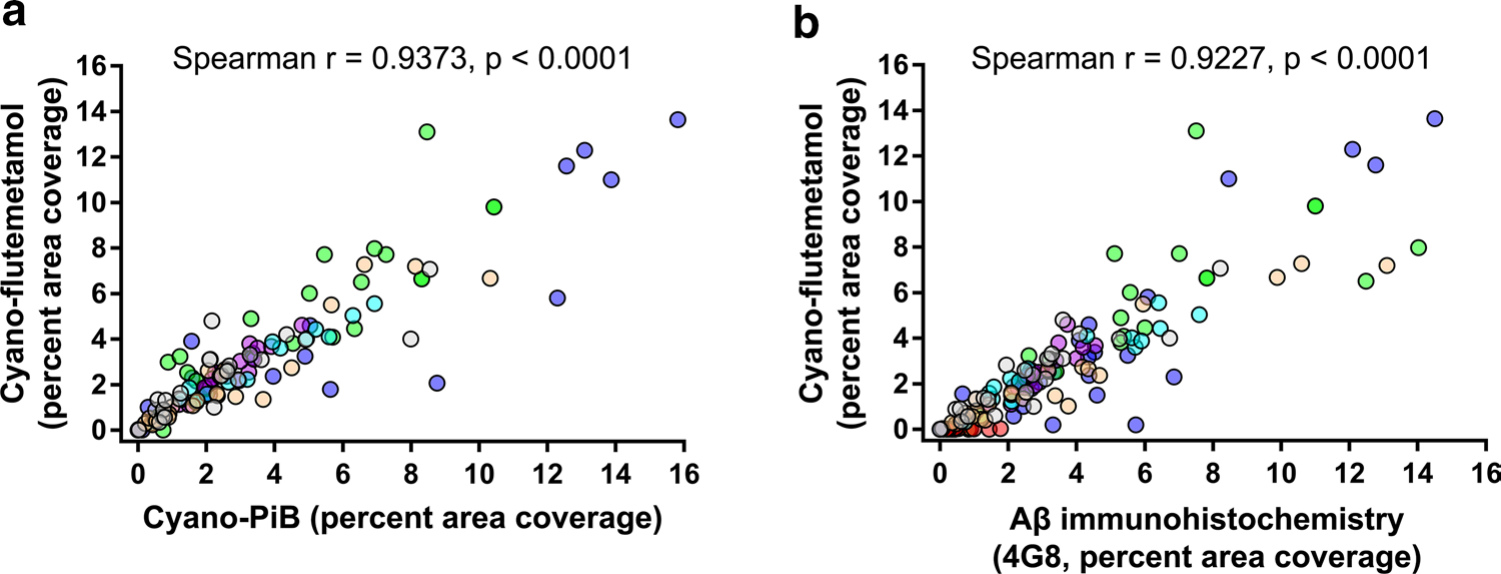
Correlation analyses of cyano-flutemetamol with cyano-PiB (**a**) and Aβ immunohistochemistry (**b**, antibody clone 4G8) total plaque load values (expressed as percent area coverage of microscopic fields) quantified in sections from 25 cortical and subcortical regions [17] from seven AD cases (#6, #10, #12, #23–25, #27, Table 1). Cyano-flutemetamol- and cyano-PiB-labeled plaque load values correlated (**a**, Spearman *r* = 0.9373, *p* < 0.0001) as did cyano-flutemetamol-labeled and Aβ-immunoreactive plaque load values (**b**, Spearman *r* = 0.9227, *p* < 0.0001). Each case is distinguished by a unique color

**Fig. 6:**
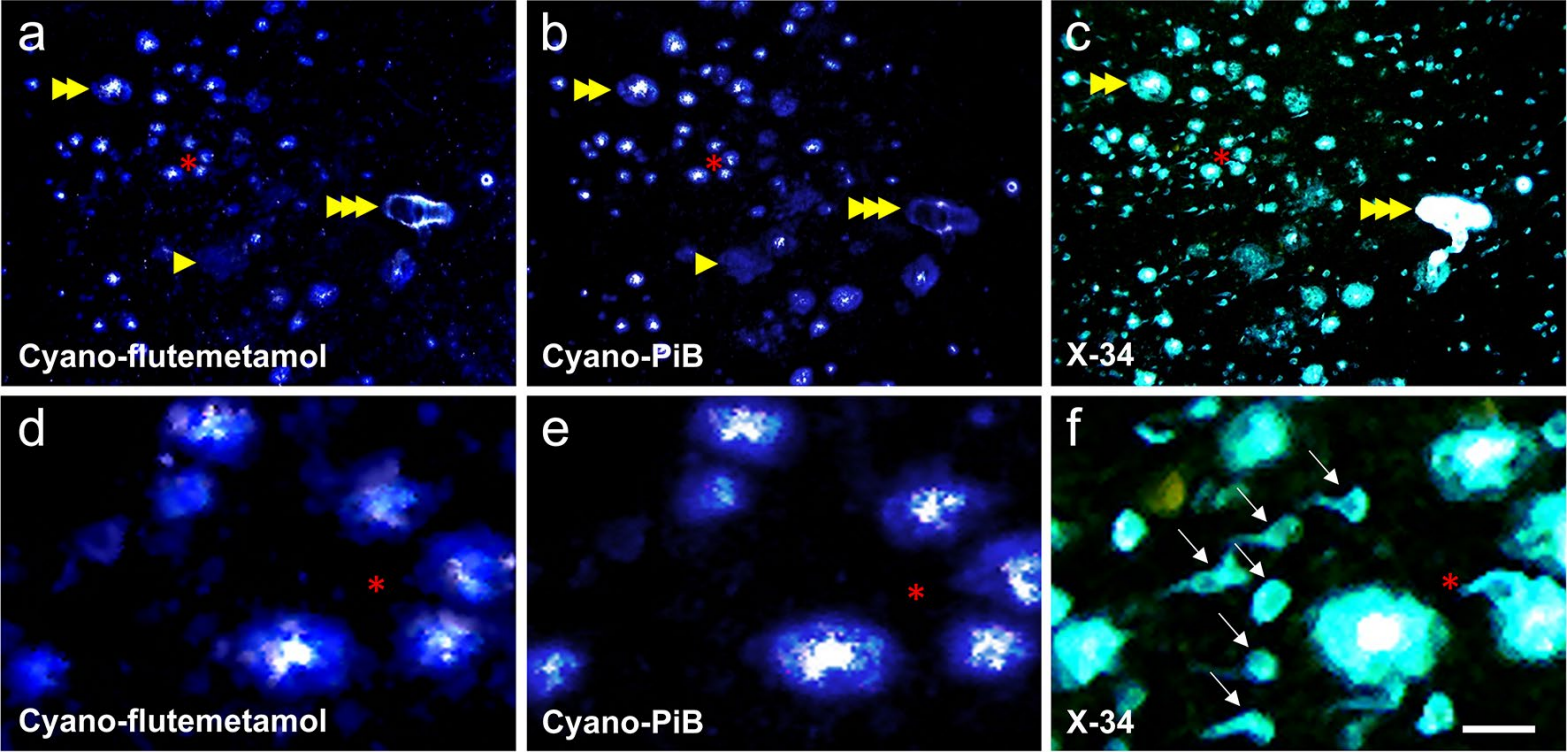
Sequential labeling of a single tissue section (thickness = 40 µm) of the frontal cortex from case #23 (AD) using cyano-flutemetamol, cyano-PiB, and X-34 histofluorescence. Cyano-flutemetamol (**a**, **d**) and cyano-PiB (**b**, **e**) label the same cored and diffuse plaques as well as cerebral vascular Aβ deposits also labeled with pan-amyloid binding dye X-34 (single arrowhead marks diffuse plaques, double arrowhead marks cored plaques, and triple arrowhead marks cerebral vascular Aβ deposits), but do not label X-34-positive neurofibrillary tangles (six neurofibrillary tangles are indicated by arrows in **f**). Higher magnification images (**d**–**f**) are from the area marked by a red asterisk in **a**–**c**. Scale bar = 100 µm (**a**–**c**), 25 µm (**d**–**f**)

For high-resolution fluorescence microscopy evaluations of flutemetamol and PiB, the 6-hydroxy group was replaced with a 6-cyano group (Fig. 3). Cyano-labeled compounds had strong absorption bands between 330 and 390 nm and emission bands between 390 and 450 nm. Competition binding assays using AD tissue homogenate showed that Ki (inhibition binding constant) values of cyano-flutemetamol (5.1 ± 0.5 nM) and cyano-PiB (5.2 ± 0.4 nM) were similar to flutemetamol (5.3 ± 0.5 nM) and PiB (3.8 ± 0.6 nM), respectively (Fig. 3). Compound binding curves are available in Supplemental Fig. 1. In sequential 8 µm thick tissue sections of frontal cortex from AD cases, cyano-PiB and cyano-flutemetamol labeled cored plaques and diffuse plaques in a pattern similar to that seen using Aβ immunohistochemistry, and neuritic plaques as seen on Bielschowsky silver stained sections (Fig. 4). In sections from multiple neocortical and subcortical regions from seven AD cases, percent area covered with cyano-flutemetamol-labeled plaques correlated strongly with percent area covered with cyano-PiB-labeled plaques (*r* = 0.93, *p* < 0.0001) and Aβ-immunoreactive plaques (*r* = 0.92, *p* < 0.0001) (Fig. 5). Cyano-flutemetamol and cyano-PiB labeling of plaques and cerebral vascular deposits closely matched the labeling of these structures with X-34, but X-34-labeled neurofibrillary tangles were not detectable with cyano-flutemetamol or cyano-PiB when these stains were applied sequentially on the same tissue section (Fig. 6).

### Confocal microscopy analysis of cyano-flutemetamol and cyano-PiB fluorescence output from individual cored and diffuse plaques

**Fig. 7:**
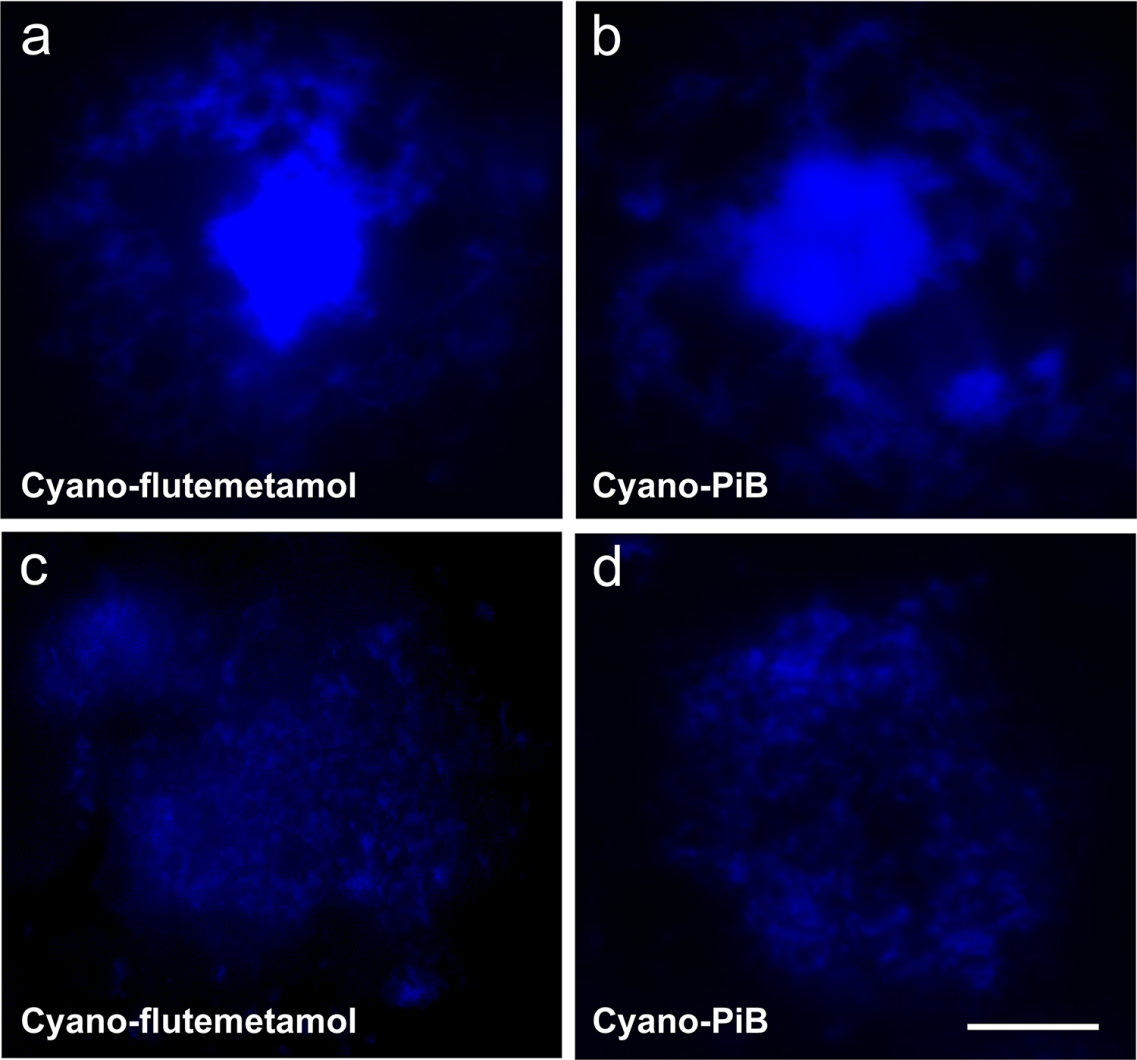
Cored (**a**, **b**) and diffuse (**c**, **d**) Aβ plaques labeled with cyano-flutemetamol (**a**, **c**) and cyano-PiB (**b**, **d**) in a thick section (~ 160 µm) from the frontal cortex from case #28 (AD, Table 1). Each image was taken from a stack of confocal images that were acquired through the full volume of each plaque (0.11 µm step size), and it represents a cross-section through the center of each plaque, illustrating the brightly labeled core of cored plaques and the uniform intensity of labeling throughout diffuse plaques. Scale bar = 12 µm

**Fig. 8:**
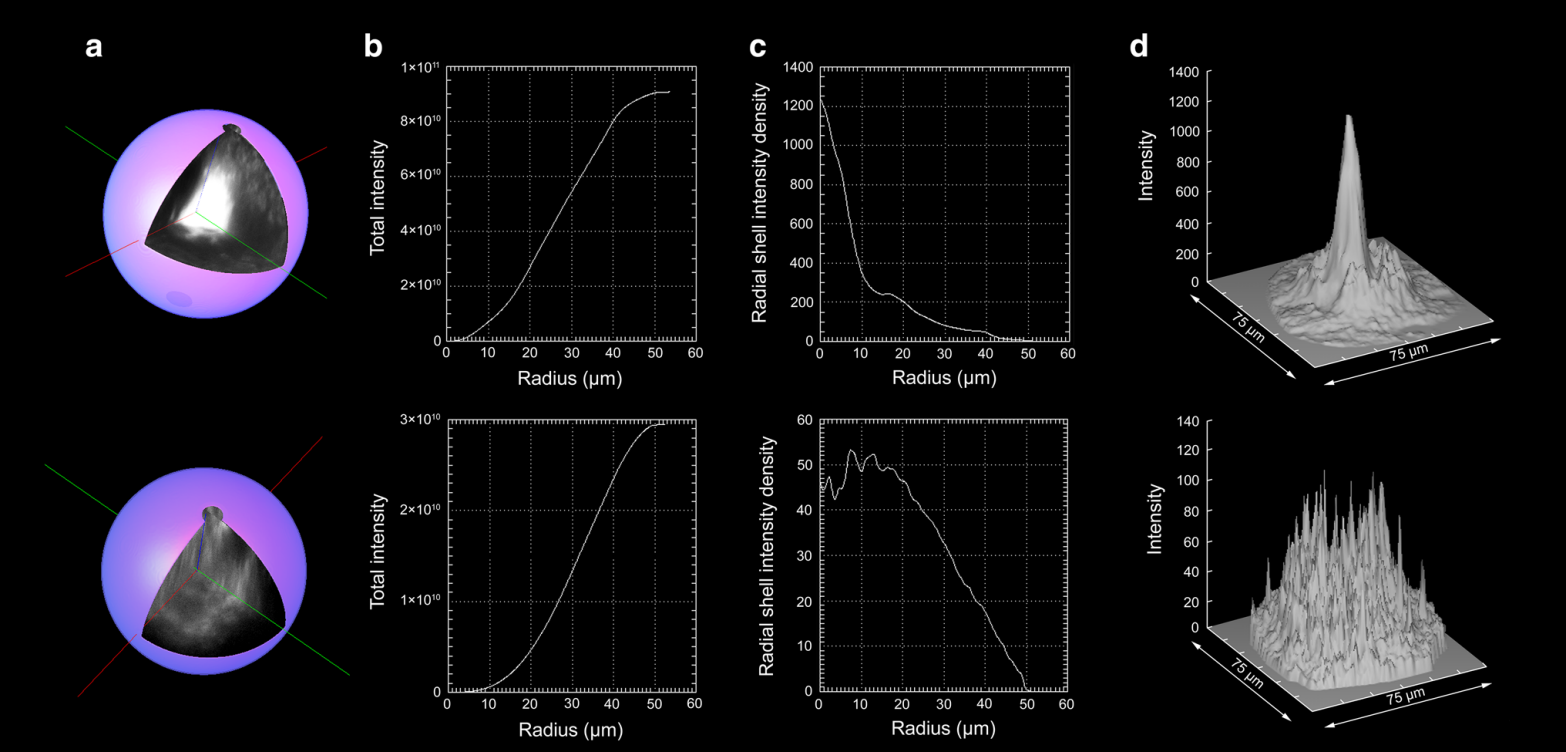
Total fluorescence output analysis of a cyano-flutemetamol-labeled cored plaque (upper row) and a diffuse plaque (lower row) imaged using confocal microscopy in thick tissue sections (~ 160 µm) from the frontal cortex from case #28 (AD, Table 1). Three-dimensional reconstructions of individual plaques (**a**) were created from confocal image stacks, and total integrated intensity plots (**b**, **c**) were generated by progressively creating shells from the center of each plaque to the periphery. Surface plots of fluorescence intensity for a cored plaque and a diffuse plaque of a similar volume are shown in **d**. Cored plaques and diffuse plaques of similar sizes gave integrated intensities of 9 ± 2.3 for cored plaques and 2.8 ± 3.1 for diffuse plaques indicating that florescence intensity of a cored plaque roughly equals fluorescence output from three diffuse plaques of the same volume

Confocal microscopy stacks of 0.11 µm-thick planes were obtained through entire volumes of individual cored plaques and diffuse plaques labeled using cyano-flutemetamol and cyano-PiB (representative examples of cross sections through the middle of each plaque type are shown in Fig. 7). Three-dimensional rendering of plaques was achieved by starting with a voxel at the center of each plaque and progressively describing shells outward to each plaque’s periphery (Fig. 8a), integrating the fluorescence intensity density throughout the shelling procedure to obtain intensity density curves (Fig. 8b, c). Cored plaques and diffuse plaques of similar sizes gave integrated intensities of 9 ± 2.3 and 2.8 ± 3.1, respectively, suggesting that in vivo diffuse plaques would need to be present at three times the frequency of the cored plaques of similar size to give comparable [^18^F]flutemetamol signal. Similar results were obtained by cyano-PiB fluorescence analysis (not shown) in tissue sections adjacent to those used for cyano-flutemetamol analysis.

### Integrated density analysis of cyano-flutemetamol and cyano-PiB

**Fig. 9:**
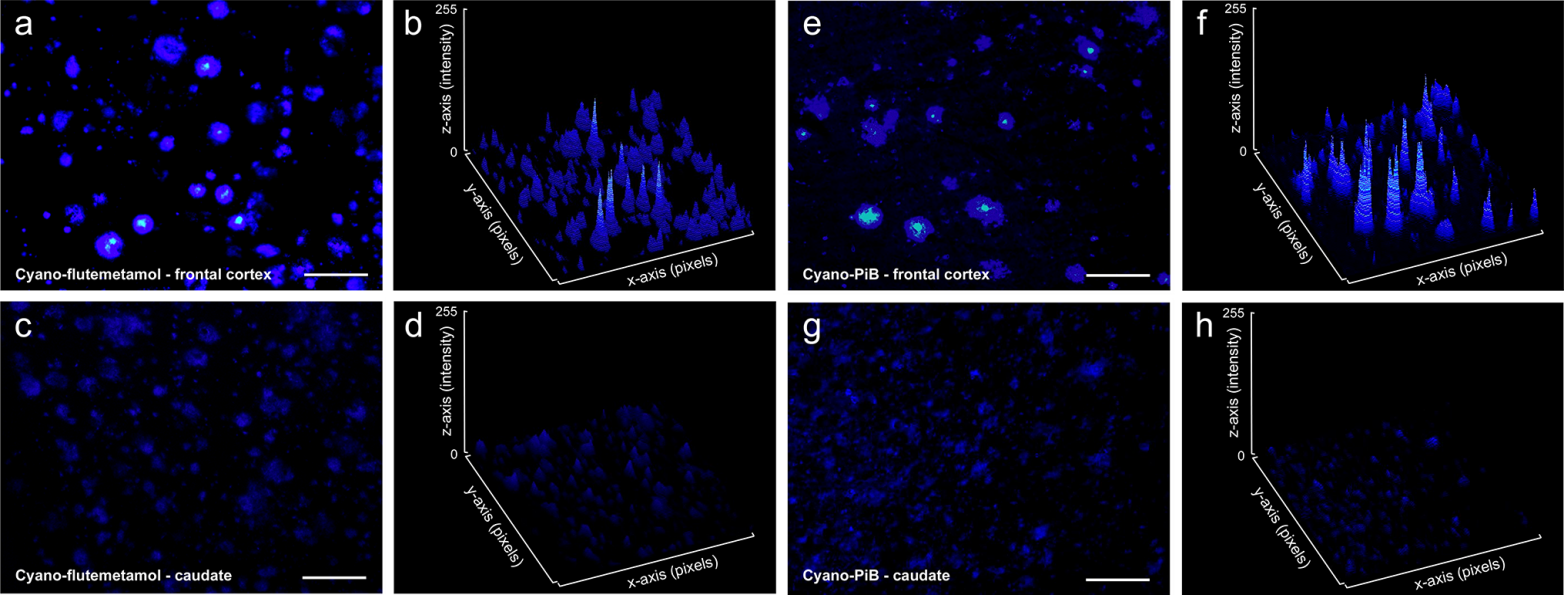
Cyano-flutemetamol (**a**–**d**) and cyano-PiB (**e**–**h**) histofluorescence in frontal cortex (**a**, **b** and **e**, **f**) and caudate (**c**, **d** and **g**, **h**) tissue sections (thickness = 40 µm) from case #24 (AD, Table 1). Percent area coverage of cyano-flutemetamol-labeled plaques was comparable in frontal cortex (9.4%) and caudate (9.8%). Similarly, percent area coverage of cyano-PiB-labeled plaques was comparable in frontal cortex (8.4%) and caudate (8.1%). Surface plots of cyano-flutemetamol (**b**, **d**) and cyano-PiB (**f**, **h**) illustrate fluorescence intensity (*z* axis) and area (*x*, *y* axes) of plaques within each microscopic field in two regions. When percent area and fluorescence intensity are integrated, resulting values differ between the two regions for both cyano-flutemetamol (**b** frontal cortex = 12,704; **d** caudate = 1188) and cyano-PiB (**f** frontal cortex = 10,069; **h** caudate = 771). Scale bar = 100 µm

**Fig. 10:**
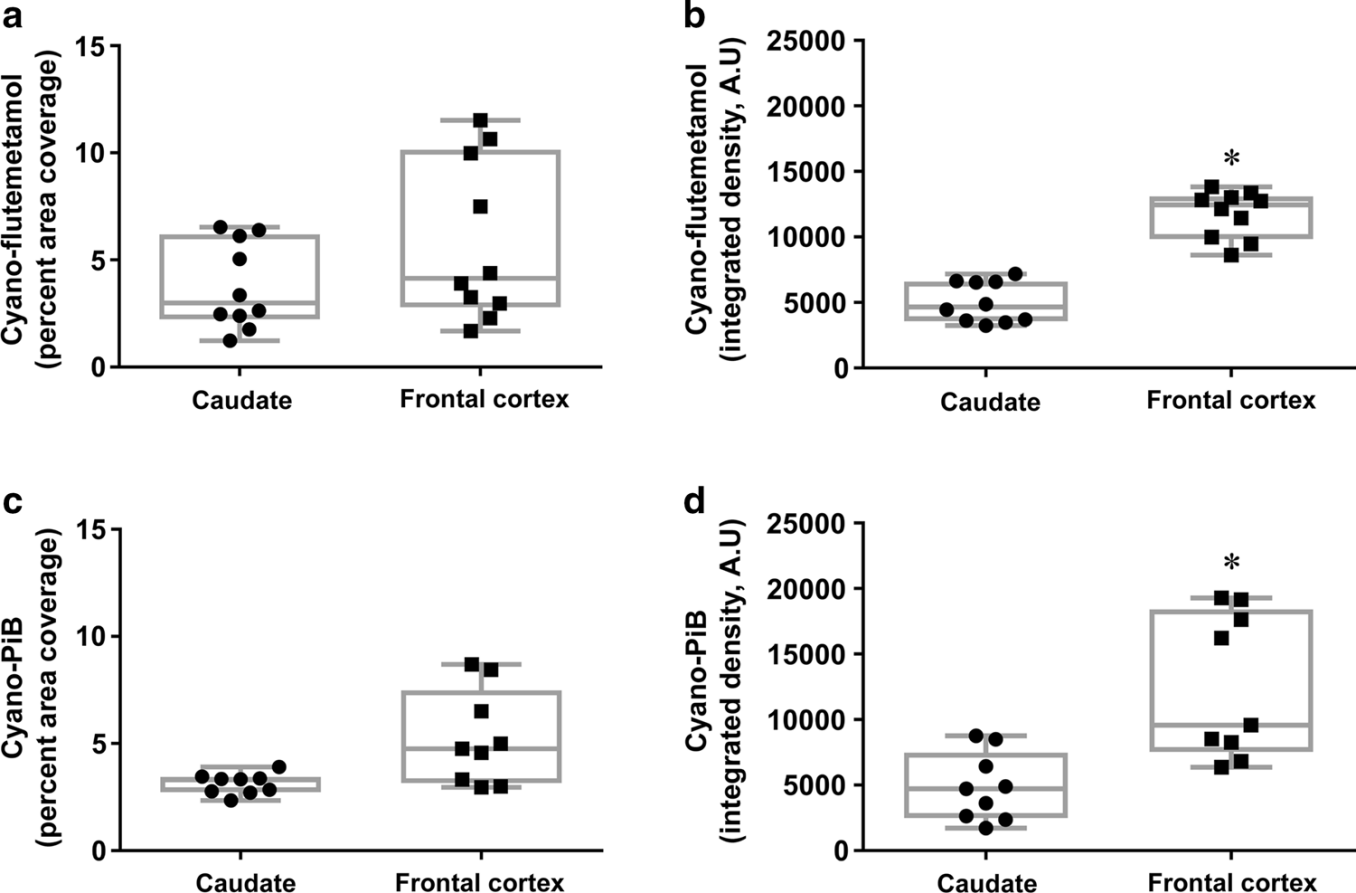
Box plots of cyano-flutemetamol- (**a**, **b**) and cyano-PiB- (**c**, **d**) labeled pathology load values obtained from microscopic images of the caudate and frontal cortex from ten AD cases (#10, #22–30) and expressed as percent area coverage (**a**, **c**) or integrated density measures (**b**, **d**). *p < 0.05 (Wilcoxon rank-sum test)

Qualitatively, cored plaques exhibit brighter fluorescence than diffuse plaques when both plaque types are labeled with either cyano-flutemetamol or cyano-PiB. This difference is clear in the example where cyano-flutemetamol-labeled plaques in the frontal cortex from AD cases (mixed deposits with mostly cored plaques, Fig. 9a) are compared to the caudate nucleus from AD cases (a region with mostly diffuse plaques, Fig. 9c). This difference can be visualized when fluorescence intensity for each plaque type is graphed on the z-axis of a microscopic field (Fig. 9b, d). Similar results were observed for cyano-PiB (Fig. 9e–h). The influence of plaque type (fluorescence intensity) and size (area covered) on overall signal assessed by measuring integrated density for cyano-flutemetamol and cyano-PiB demonstrated that for two brain regions with similar percent area coverage of plaques, but with different proportions of cored plaques and diffuse plaques, the region with a greater proportion of cored plaques yielded a greater integrated density value for each ligand (Fig. 10).

## Discussion

Amyloid PET radiopharmaceuticals flutemetamol and PiB interact with Aβ fibrils in diffuse plaques, cored plaques (with or without neuritic pathology), and cerebral amyloid angiopathy (CAA) and do not bind to neurofibrillary tangles. The relative contributions of diffuse and cored plaques to [^18^F]flutemetamol and [^11^C]PiB PET retention are not known but are likely influenced by both the extent (size and frequency) of plaques and packing densities of Aβ fibrils within plaques. In neocortical regions of AD brains, diffuse and cored plaques are typically intermixed, thus in vitro binding assays with ^3^H-labeled compounds are limited in their ability to discern ligand binding to each plaque type. To overcome this limitation, we designed a histopathology study to analyze flutemetamol and PiB in diffuse plaques separately from cored plaques in post-mortem brain tissue sections at the high resolution of conventional and confocal fluorescent microscopy. Due to the low endogenous fluorescence of native flutemetamol and PiB compounds, we modified them by replacing the 6-hydroxyl group with a 6-cyano group. This modification only slightly altered each ligand’s binding affinity as determined in AD cortical tissue homogenate (see Supplemental Fig. 1) and is not expected to significantly change their interactions with Aβ fibrils.

The current study demonstrates close correspondence between cyano-flutemetamol and cyano-PiB plaque loads, as well as their strong correlation with total plaque burden determined by Aβ immunohistochemistry. This finding is further supported by our observation that [^3^H]flutemetamol and [^3^H]PiB autoradiography signals corresponded closely to Aβ plaques detected by immunofluorescence. Because autoradiography and Aβ immunofluorescence procedures were performed on separate, adjacent sections, some plaques of smaller size may not have spanned both sections. Additionally, Aβ immunofluorescence is slightly more sensitive than the autoradiography technique and appeared to reveal slightly more pathology than that observed in the autoradiograms (Fig. 1) in part due to occasional intracellular APP/Aβ immunoreactivity. It is worth noting that the scaling factor between cyano-flutemetamol and cyano-PiB positive histological plaque burdens in tissue sections analyzed in the current study is similar to that in a recently reported Centiloid analysis of paired [^18^F]flutemetamol and [^11^C]PiB scans from 74 subjects [3]. In support of the idea that both ligands’ fluorescence intensity is influenced by Aβ fibril density, our current study shows that cyano-labeled derivatives of the two ligands exhibited bright fluorescence in cored plaques, characterized by high Aβ fibril packing density in the core region, and dimmer fluorescence in diffuse plaques, characterized by loosely organized Aβ fibrils. We demonstrate that total fluorescence output from a cored plaque is approximately three times that from a diffuse plaque of the same volume. Our integrated density measures of cyano-labeled ligand signal output from plaques, calculated as a combination of fluorescence intensity (a surrogate measure of Aβ fibril packing density) and plaque area coverage showed that for brain regions with similar plaque area coverage, the region with more cored plaques yielded higher integrated density values. Thus, although cored plaques bind more ligand than diffuse plaques, high densities of the latter plaque type could generate significant flutemetamol and PiB retention and explain the robust amyloid PET signal in the striatum (a brain region where diffuse plaques predominate) in cases interpreted to be positive for Aβ plaques.

The proposed contribution of diffuse plaques to amyloid PET retention signal is in agreement with false positive results reported in several clinicopathological studies where brain regions determined as positive on visual PET reads had low/subthreshold levels of neuritic plaques by Bielschowsky silver stain, but significant amounts of diffuse plaques on post-mortem analyses by Aβ immunohistochemistry [6, 8, 13, 16, 36, 38]. Accordingly, in a validation study of [^18^F]flutemetamol PET, a false positive result by CERAD neuropathology criteria [30] turned into a true positive according to the 2012 NIA-AA neuropathology criteria that takes into account all types of Aβ-immunoreactive plaques [16]. These results are also in agreement with a study by Beach and colleagues where striatal positivity on [^18^F]flutemetamol PET scans was observed in the presence of moderate-to-frequent densities of diffuse Aβ plaques in this brain region [4]. Collectively, our results indicate that amyloid PET may best reflect neuropathology measures by quantitative Aβ immunohistochemistry which labels all types of Aβ plaques.

There are several limitations to the current study. AD cases were all the sporadic form, so certain plaque types abundant in autosomal dominant AD and rarely detected in sporadic AD, such as cotton wool plaques, were not evaluated. Direct correlations of pathology data with amyloid PET were not possible since we used archived brain tissue samples from cases without ante-mortem PET imaging data. The binding affinity of other amyloid PET tracers such as the stilbenes florbetaben and florbetapir to cored plaques and diffuse plaques may be different and it would be of interest to assess using methodologies similar to those described herein. In addition, the contribution of vascular Aβ deposits to ligand retention remains to be explored. Collectively, these and additional future studies will provide valuable information regarding the ability of amyloid PET tracers to detect different forms of Aβ deposits, with direct implications for clinicopathological studies using these ligands.

## Electronic supplementary material

Below is the link to the electronic supplementary material.

Supplemental Figure 1. Binding curves for PiB, flutemetamol and their cyano-labeled derivatives. (TIF 27617 kb)
