# Supplementary figures and images for: Post-mortem analyses of PiB and flutemetamol in diffuse and cored amyloid-β plaques in Alzheimer’s disease
Source: Acta Neuropathol. 2020 Aug 9;140(4):463–76. doi: 10.1007/s00401-020-02175-1 (PMC7498488; doi:10.1007/s00401-020-02175-1)

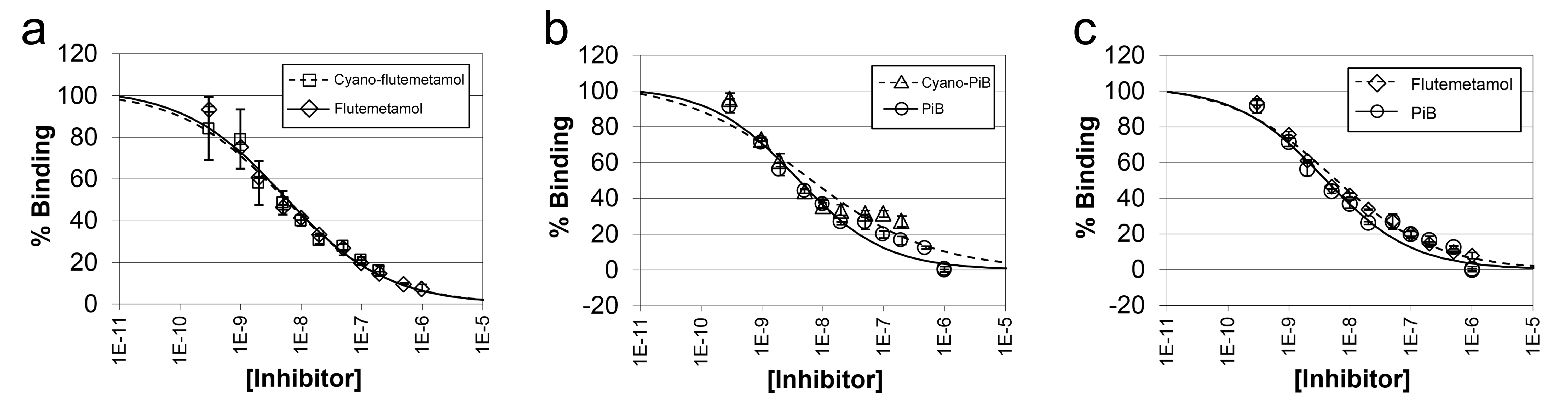

Supplement: Supplementary file 1 — Supplemental Figure 1. Binding curves for PiB, flutemetamol and their cyano-labeled derivatives. (TIF 27617 kb) [file 401_2020_2175_MOESM1_ESM.tif]
